# Contrast‐Enhanced Ultrasound (CEUS) Reveals Perfusion of Human Bone in Acute Fracture Healing: A Pilot Study

**DOI:** 10.1002/jor.70100

**Published:** 2025-12-13

**Authors:** D. Cadoux‐Hudson, M. Thomas, J. Hurst, R. Schranz, A. Gerrish, K. Wallace, D. Warwick, D. Carugo, E. Stride, S. Tilley, N. D. Evans

**Affiliations:** ^1^ Bone and Joint Research Group University of Southampton Southampton UK; ^2^ Department of Trauma and Orthopaedic Surgery University Hospital Southampton Southampton UK; ^3^ Southampton Centre for Biomedical Research University Hospital Southampton Southampton UK; ^4^ GE Healthcare Niskayuna New York State USA; ^5^ Botnar Research Institute University of Oxford Oxford UK

## Abstract

Bone fractures are common injuries with reported non‐union rates of up to 9%. Current treatments for non‐union include surgery with complication rates of up to 17% and significant costs. Microbubbles are used clinically in ultrasonography as contrast agents and have been shown to deliver therapeutics to desired locations by noninvasive stimulation of cavitation using extracorporeal ultrasound in preclinical studies. Contrast enhanced ultrasound (CEUS) has been used to determine the cause of established fracture non‐unions, though this is not in widespread use clinically. This pilot study aimed to test the hypothesis that peripherally injected microbubbles are detectable in acute fracture sites. Adult patients (18–75 yrs) with acute humeral shaft fractures were recruited to undergo CEUS within 28 days of injury. They underwent peripheral injection of SonoVue microbubbles with ultrasound imaging. B‐mode and contrast‐mode videos were collected and time‐intensity curve analysis was used to assess for the presence of microbubbles at the fracture site. Ten patients were recruited, 8 underwent humeral shaft fracture scans with 7 analysed. All fracture sites demonstrated increased contrast signal following injection. The wash‐in volume of microbubbles was greater than the wash‐out volume in all cases, with a mean difference of 1.4 × 10⁻⁵ (± 1.7 × 10⁻⁵) (*p* = 0.015 Wilcoxon Test). There was a trend toward decreasing PI and TtP with fracture age, though not statistically significant (R² = 0.44, *p* = 0.1; R² = 0.24, *p* = 0.26, respectively). This pilot study demonstrates that commercially available microbubbles perfuse acute fractures in ultrasonographically detectable quantities using commercially available equipment.

## 1 Introduction

Traumatic bone fractures are a common condition, and although the vast majority go on to clinical and radiological union, a proportion (1.9%–9%) will ultimately result in a non‐union [1, 2]. This is characterised by a failure to progress to union without further intervention. Current treatment of fractures aims to maintain long term function by achieving union. This involves either non operative management with immobilisation, or surgical intervention to maintain reduction and optimise the fracture healing process [3]. There are few other treatment options available to minimise the risk of developing a non‐union following a fracture. Current treatment modalities for non‐unions include surgical treatment such as fixation, bone grafting (autograft or allograft) through to arthroplasty procedures or even amputations. These treatments carry significant cost implications with 2006/2007 cost estimates ranging from £15566 to £17200 per patient for humeral and femoral fracture non‐unions respectively in the UK [4]. A 2013 analysis of surgical treated tibial fractures in the US concluded that non‐union increased treatment costs by $13870 [5].

There are several factors that contribute to a non‐union developing. These can either be due to factors specific to the fracture, or patient related factors. Examples of fracture related factors include anatomical location, often related to the nature of the blood supply in that area such as the proximal scaphoid which has a tenuous blood supply and a very high rate of non‐union (approx. 34%) [6]. Another example of a fracture related factor is the degree of energy of the injury and subsequent level of disruption to the soft tissues surrounding the fracture such as the periosteum and subsequent blood supply [7]. Non fracture related factors include smoking which is postulated to stimulate vasoconstriction and therefore compromise blood supply [8]. Diabetes increases the risk of reduced perfusion due to small vessel disease and therefore inhibit healing. Non steroidal anti inflammatory drugs are postulated to inhibit bone healing via the cyclooxygenase pathway resulting in a reduction in callus formation [9]. It is clear that bone healing is dependent on an adequate perfusion and the management and assessment of bone healing is dependent on this.

Despite this it remains challenging to measure blood supply in fractures, either before, or after the establishment of non‐union, and is not routinely undertaken in clinical practice. Methods that have been investigated include, the in vivo use of positron emission tomography (PET) scanning of rat femurs using radiolabelled fluoride to assess for the development of non‐unions [10] demonstrating that fractures with reduced blood supply were more likely to develop non‐unions. In clinical studies labelled water contrast PET scanning has demonstrated an increase in the perfusion of fractured tibiae when compared to normal contralateral limbs within 24 h of fracture with a higher increase in undisplaced compared to displaced fractures [11]. The use of both plain or B mode ultrasound to assess for bone healing has been demonstrated in a clinical setting as a reliable indicator of the progression of bone healing [12], however this is not used in widespread clinical practice and does not give an indication of the underlying blood supply of the fracture. Doppler ultrasound scanning utilises the effect of fluid passing through a sound wave and the resultant change in frequency of the reflected sound to determine the direction and rate of blood flow. Doppler has been used to demonstrate the presence of neovascularisation in fracture sites, but relies on the presence of vessels that are visible and is sensitive to the 2 dimensional nature of Doppler scanning and therefore blood supply can underestimated using this method [13].

Another technology which has significant potential in tracking vascularity in bone fractures is CEUS. CEUS depends upon the injection of a contrast agent – usually a microbubble (MB) – the perfusion of which can subsequently be imaged noninvasively from outside the body using an ultrasound imaging device. MBs are widely used in medical imaging as a contrast medium in ultrasonography, they have a good safety profile and are in use worldwide [14]. They generate harmonic responses under ultrasound conditions, whereby the reflected signal is a multiple of the input signal due to the harmonic cavitation of the microbubbles. Cavitation is the process whereby the MB vibrates at a frequency greater than its driving frequency. Standard B mode imaging relies on linear responses of tissues whereby the reflected signal is at the same frequency as the input signal and the image generated is a function of the amplitude of the returned signal. The characteristics of MBs in contrast imaging allow for fluids and tissues that would not normally generate a linear response to be imaged in greater detail.

MBs have been demonstrated to effectively show perfusion of fracture sites in canine fractures models and to demonstrate the vascularity of normal bone healing [15]. MBs have been used clinically in the fracture setting as a means of assessing established non‐unions. Haubruck et al. used Contrast Enhanced Ultrasound (CEUS) to assess for blood flow in a small cohort of patients (*n* = 13) [16]. Fisher et al. used CEUS to differentiate between infected and atrophic non‐unions, and as a marker of healing post operatively [17]. In these studies patients underwent CEUS imaging of fracture sites demonstrating increased microbubble intensity in patients who had an infected non‐union in comparison to those who had an atrophic non infected non‐union. CEUS has also been used to assess for vascularity in the rotator cuff muscles of the shoulder in healthy volunteers and in those who have undergone surgical repair. An increase in blood supply with both exercise and surgery measured by contrast enhanced ultrasound was reported [18, 19]. The use of this imaging technology in the acute fracture setting has not been explored, and therefore the viability of using microbubbles for therapeutic purposes in the setting of acute trauma to accelerate healing and prevent the development of non‐union in at risk patients is uncertain. This phenomenon has been demonstrated clinically using three‐dimensional contrast imaging but not with CEUS. Contrast enhanced imaging has demonstrated that non‐unions maintain a blood supply (albeit small in comparison to an infected non‐union) long after the initial peak blood supply.

All these studies used CEUS to distinguish between classes of non‐union in tibiae, rather than in using CEUS at early stages of bone healing as a means of establishing a method that might be used to predict non‐union. There are no reports of the use of contrast enhanced ultrasound in the acute fracture setting.

The exact nature of how blood supply and the ability to observe this using CEUS changes over time is unknown. There is little literature concerning the ability for peripherally injected microbubbles to reach acute fracture sites in clinical scenarios, nor any description of associated flow rates and signal intensities.

The aims of this pilot study are to demonstrate detectable levels of microbubbles within an acute fracture in a clinical setting and to determine the volume and intensity of that signal relative to time from injury.

## 2 Methods

### 2.1 Ethics

The study received ethical approval from the Health Research Authority, National Health Service, UK with protocol number 60771 and Research Ethics Committee reference 20/NS/0032 granted to the University of Southampton.

### 2.2 Patient Selection

Patients with a humeral shaft fracture were prospectively recruited to this study over an 18‐month period. Inclusion criteria were age between 18 and 75, isolated injury, able to consent to the study, within 28 days of injury and no contraindications to SonoVue (Bracco, Italy).

- Hypersensitivity to the active substance(s) or to any of the excipients.
- Right‐to‐left shunts.
- Severe pulmonary hypertension (pulmonary artery pressure > 90 mmHg).
- Uncontrolled systemic hypertension.
- Adult respiratory distress syndrome.

Patients were diagnosed with plain film X‐rays and were treated with a removable brace.

### 2.3 Radiological Evaluation

Patients were diagnosed in the emergency department. They were recruited from the referrals to the Trauma and Orthopaedic Department at University Hospitals Southampton via the Emergency Department and local Urgent Care Centres. Fractures were assessed radiologically using orthogonal views as to whether they were a true shaft of humerus fracture. Any fractures that extended into the surgical neck of the humerus were excluded. Any fracture which had been treated surgically was also excluded.

### 2.4 Contrast‐Enhanced Ultrasound (CEUS) Assessment

All patients who were recruited and consented to imaging underwent CEUS assessment provided they were within 28 days of injury. This was performed by the same consultant radiologist at University Hospitals Southampton. A GE Logiq E19 Ultrasound Machine was used in conventional B mode imaging to assess the fracture site with the brace removed. The largest fracture gap was identified in a longitudinal plane. Once this was identified dual view contrast mode was enabled. A 2.4 mL bolus of SonoVue, a widely available commercial preparation of sulphur hexafluoride microbubbles dispersed in normal saline, was injected into a vein in the contralateral ante cubital fossa using a minimum of a 20‐gauge cannula. This was followed with 10 mL 0.9% (w/v) saline solution flush to clear any remaining microbubbles from the venous cannula. Video recording was commenced before injection of the contrast agent. Recording continued until the observed peak of signal intensity had dissipated back towards the baseline observed before injection based on visual assessment by the radiologist. This method was preferred over a time determined method to ensure capture of the observed wash out period.

The video clip underwent post‐processing using the proprietary software available on the GE imaging device. The Region of Interest (ROI) was identified using B mode imaging, this was drawn over the largest visualised fracture gap and customized to exclude areas beyond the fracture callus which could potentially interfere with the signal such as fracture fragments, intact bone or fascia [17]. This area subsequently underwent time intensity curve analysis. The data were smoothed using a 50‐point smoothing protocol to provide a more representative sample. Data outputs included peak intensity (PI) in arbitrary units, area under the curve (AuC) in arbitrary units, and Time to Peak (TtP) measured in seconds.

### 2.5 Statistical Analysis

Further processing was carried out using Microsoft Excel and Prism graphing software. Time since fracture was plotted against the variables mentioned above. R^2^ values were calculated by linear regression using Prism. Wilcoxon Test was used to compare WiAUC to WoAUC with a *p* value on 0.05 used to determine significance.

## 3 Results

### 3.1 Patient Characteristics

Ten patients who had sustained acute humeral shaft fractures were recruited of whom eight underwent contrast enhanced ultrasonography and analysis. The radiographs for the seven fractures are shown in Supporting Figure 1. Two patients were excluded as they were more than 28 days following injury by the date of scanning. One of the patients who underwent scanning the proprietary data files were lost from the device due to a technical issue and were analysed separately, and therefore was not included in the grouped analysis. The average age of those who underwent scanning was 58.9 ± 9.2 years, 43% of whom were female (see Table 1).

**Table 1:** Details of patients who underwent scanning and whose data was analysed.

Patient number	Gender	Age	Time from fracture (days)
1	F	68	5
2	M	64	9
3	F	54	12
4	M	41	13
5	M	65	18
6	F	57	23
7	M	63	28
Mean (SD)		58.9 (9.2)	15.4 (8.1)

### 3.2 Microbubble Perfusion is Detectable in Acute Bone Fracture

**Figure 1:**
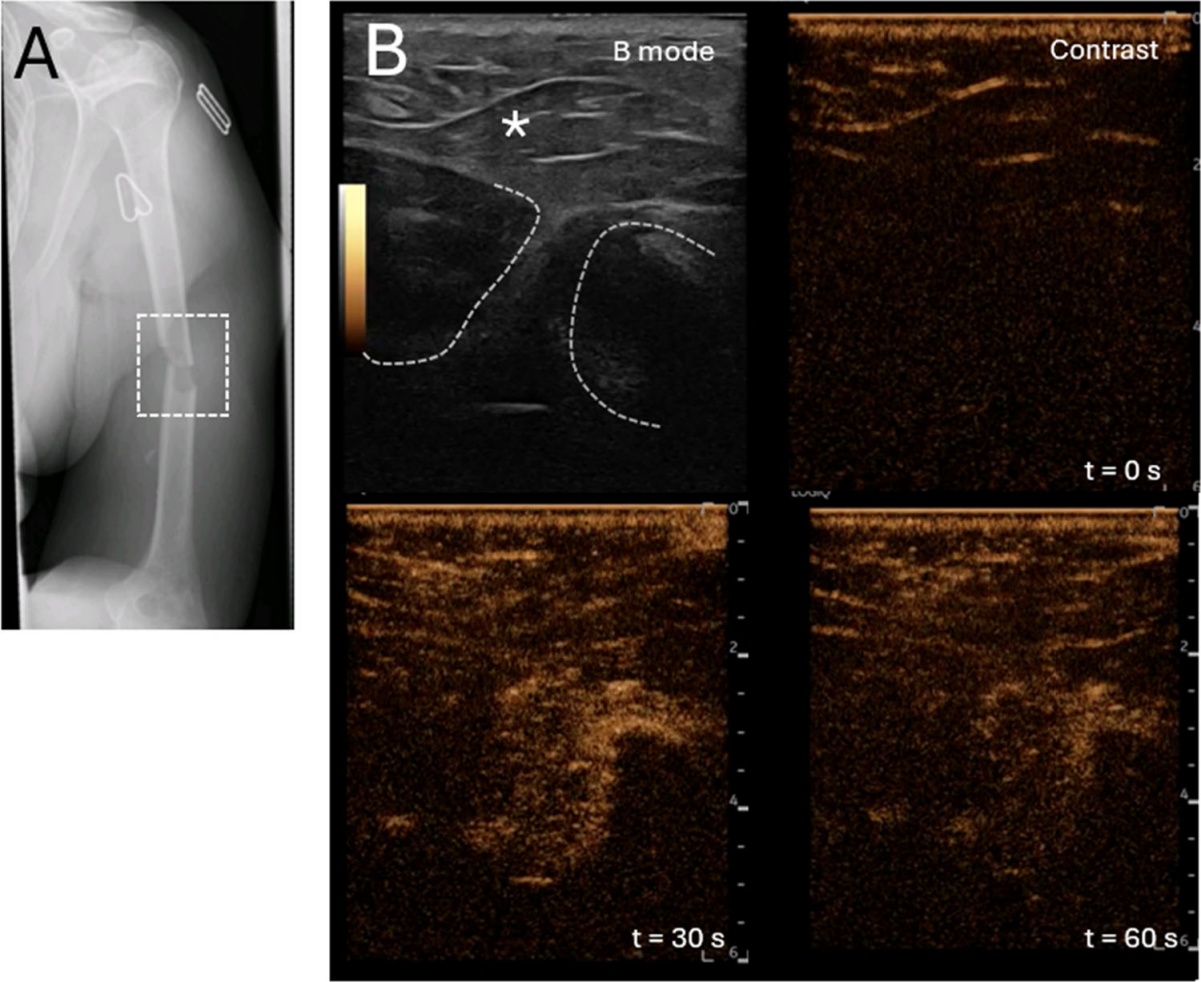
Contrast agents can be detected in humeral fracture gaps. (A) Patients were recruited with displaced fractures of the humerus as identified on radiographs. (B) The fracture gap was identified as a discontinuity in the bone, as shown in the B mode image with the bone edges indicated by dotted white lines and superficial muscle by *. Contrast perfusion of the fracture gap was evident as an increase in contrast over a period of 100 s of seconds post perfusion.

To test the hypothesis that microbubble perfusion of acute bone fractures could be imaged using contrast enhanced ultrasonography, eight patients were injected peripherally with Sonovue concurrent with continuous imaging of the bone fracture region. In patient 2, the displaced fracture of the humerus was first identified on X‐ray images at presentation (Figure 1A). At 5 days post fracture, was located using B mode radiography (Figure 1B, top left). The bone was located as a B‐mode dark area beneath the deep fascia. Within 20 s after initial intravenous injection there was a marked and rapid increase in signal in the area of the fracture gap and in more superficial tissues (Supporting Video 1 and Figure 1B).

**Figure 2:**
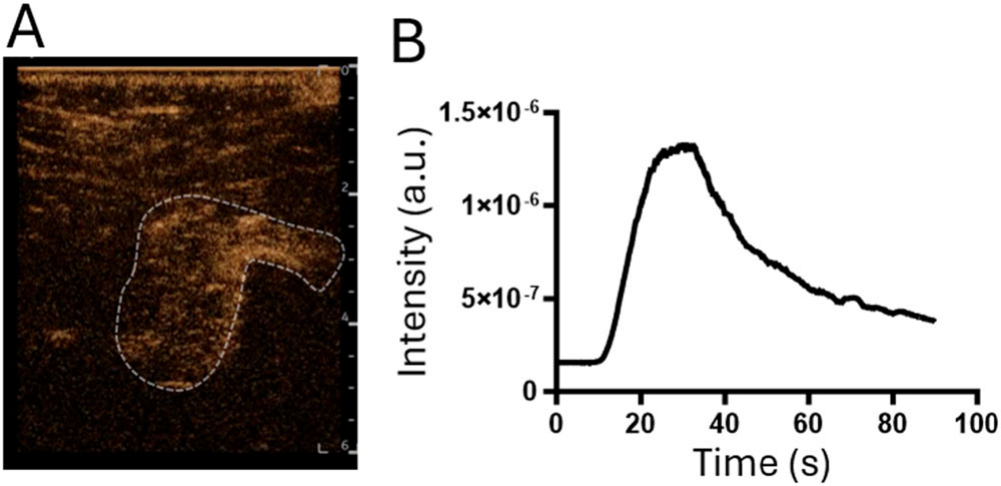
Dynamic perfusion of fracture gaps can be quantified with respect to time. (A) Fracture regions were marked with a ROI and the relative acoustic intensity was quantified with respect to time (B) showing a rapid peak ~30 s post injection and a slower wash‐out of > 1 min.

To quantify the change in contrast in the fracture area, a region of interest was manually drawn around the fracture area using GE software (Figure 2B). A time vs intensity curve was plotted, which indicated the rapid rise in signal followed by a more gradual decrease.

The same analysis was conducted on all seven patients for which data were available. Every participant demonstrated a rapid inflow of microbubbles with a slower washout period. There was some variability in the signal intensity within the fracture callus and fracture site identified. This is likely to be due to variabilities in the blood supply within the callus in addition to the variability of the ultrasound reaching areas of the ROI due to the surrounding bone. (Supporting Videos [Link], [Link] and Supporting Figures [Link], [Link]). Similar data were obtained from raw files for Patient 8 (Supporting Figure 8).

**Figure 3:**
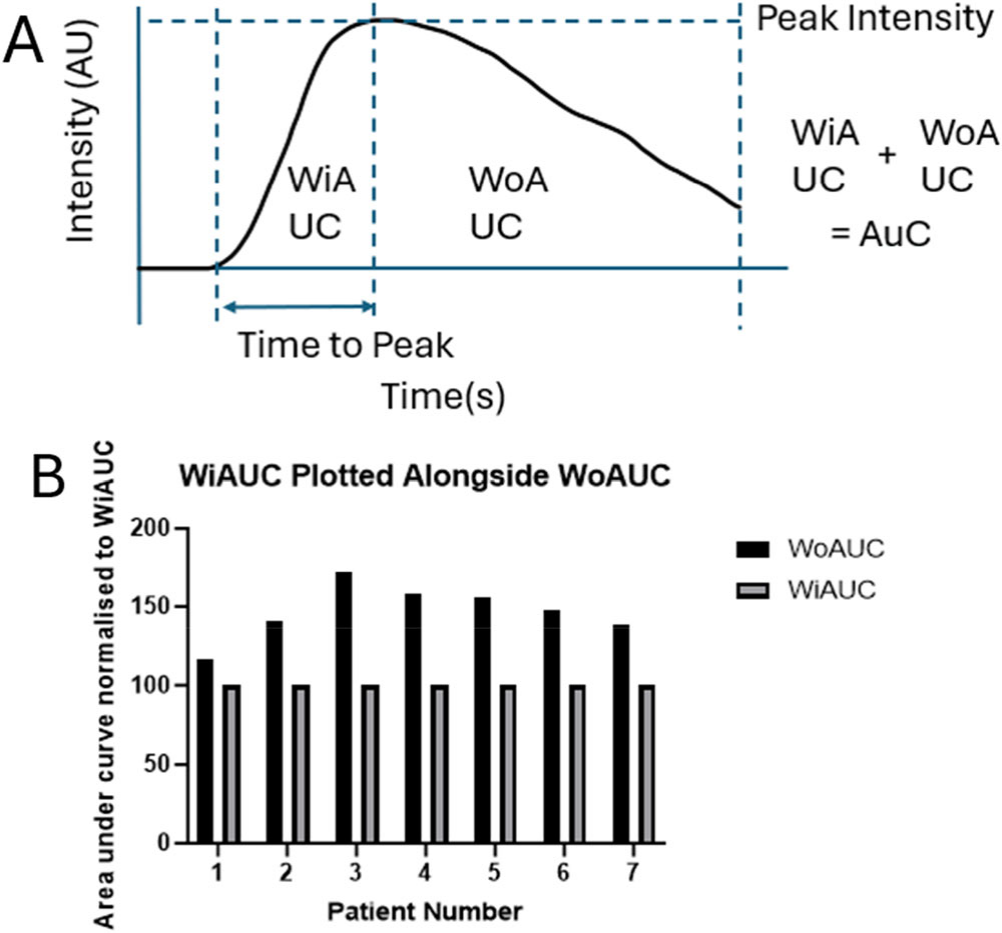
The measurements used to compare fraction perfusion by CEUS are illustrated in A, and comprise a typical time intensity curve with annotations indicating time to peak (TtP), peak intensity (PI), wash‐in area under curve (WiAUC), wash‐out area under curve (WoAUC), and area under curve (AuC). In all patients, the WoAUC was greater than WiAUC (B), indicating a slowed rate of decline in microbubble perfusion from PI compared to increase in microbubble perfusion.

To determine quantitatively the kinetics of the microbubble perfusion during bone fracture a number of parameters were measured including *peak intensity* (reflecting maximum perfusion at the region of interest; PI) *time to peak* (reflecting the time taken for the perfusion of the facture to reached maximum; TTP), *wash‐in area under the curve* (reflecting the integral of the rate until the maximum perfusion was reached; WiAUC) and *wash‐out under the curve* (reflecting the integral of the rate until the end of the video as the line trends towards zero); WoAUC) and *area under the curve* (a representation of the quantity and amplitude of interactions between the ultrasound beam and the microbubbles within the area of interest giving a representation of the overall volume of microbubbles that were present in the area); AuC. A diagrammatic explanation of these terms with respect to a typical CEUS intensity trace is shown in Figure 3C.

The mean peak intensity for all fractures (PI) was 1.95 × 10^−6^ ± 1.6 × 10^−6 ^AU with a mean TTP of 23.4 ± 7.9 s, a WiAUCof 4.7 × 10^−5^ ± 4.3 × 10^−5 ^AU and a WoAUC of 3.1 × 10^−5^ ± 2.7 ×10^−5 ^AU. In all cases the decline in perfusion of the fracture took longer that the rise in perfusion measured by CEUS, reflected in a significantly larger WoAUC compared to WiAUC with a mean difference for all patients of 1.4 × 10^−5^ ± 1.7 × 10^−5^ (47.2%; *p* = 0.015 Wilcoxon Test).

**Figure 4:**
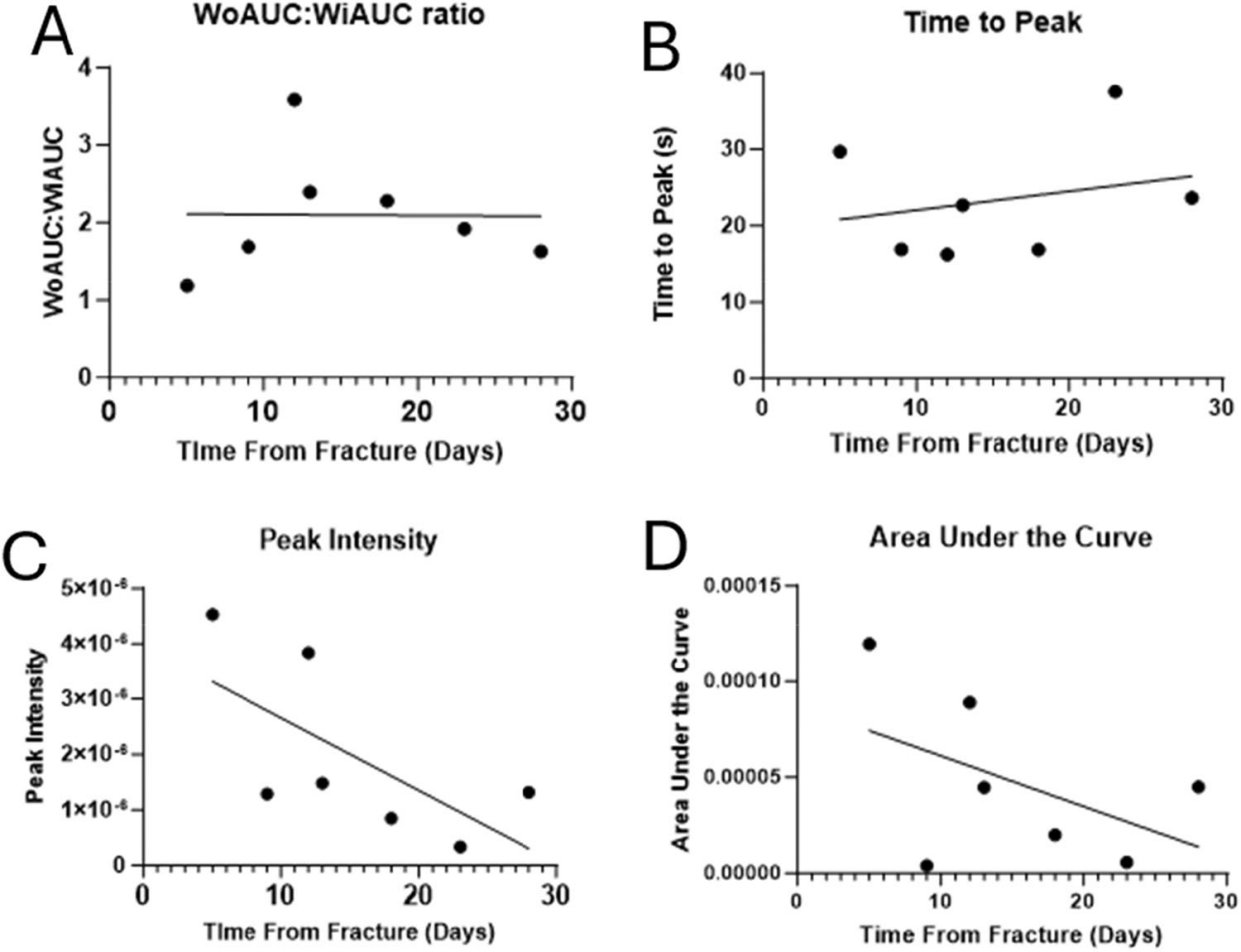
Characteristics of CEUS scans with respect to time since fracture. There was no strong relationship between with WoAUC:WiAUC ratio or Time‐to‐Peak with respect to time to fracture (A and B), but there was a downward trend for both Peak Intensity (R2 = 0.44) and AUC (R2 = 0.24) but this was not significant (*p* = 0.1 and 0.26 respectively).

To determine if there were any emerging patterns in the degree or kinetics of perfusion with respect to time since fracture, several parameters were measured with respect to time since fracture, including WoAUC:WiAUC, PI, AuC and TtP (Figure 4). There was no strong relationship between WoAUC:WiAUC or TtP and time since fracture, but there were indications of emerging relationships between PI and AUC and time to fracture, although these relationships were not significant (R^2^ = 0.44, *p* = 0.1; R^2^ = 0.24, *p* = 0.26 respectively). The graph demonstrates a reduction in the AuC with increasing time from the initial injury to scanning.

In summary these pilot data indicate that perfusion of acute human bone fractures can be detected using CEUS. More data is required to establish quantitative relationships with respect to time since fracture and perfusion‐related CEUS parameters.

## 4 Discussion

The ability for microbubbles to perfuse acute fracture sites is a key step to determine the potential for their use as a delivery agent. Although the traditional understanding of the process of fracture healing would suggest that this is possible, as far as the authors are aware, this has not been demonstrated in a clinical setting. Alternative noninvasive options such as low‐intensity pulsed ultrasound (LIPUS) exist and are clinically available, their efficacy in preventing or treating non‐unions remains controversial. This is a noninvasive method that aims to augment bone healing by generating micro movement at fracture sites. It is approved by NICE for use in the delayed union setting although with the caveat that ‘the current evidence on efficacy is inadequate in quality’. A systematic review by Schandelmaier et al. [20] has cast doubt on whether LIPUS improves patient outcomes in the delayed or non‐union setting. Systemic treatment such as teriparatide and romosuzumab have been trialled with limited success [21, 22] and are not in widespread use in clinical practice. currently, no universally accepted or consistently effective noninvasive treatments are available for patients at risk of non‐union or with established non‐unions.

In addition, MBs have additional characteristics that can be utilised to deliver a therapeutic effect in a specific region including the ability to be insonated by pulsed ultrasound resulting in stable oscillation of the MB through to inertial (unstable) cavitation at higher pressures and durations [23]. Resultant local pressure effects lead to increased cell membrane and basement membrane permeability and subsequently raised local intracellular drug concentrations [24]. Under inertial cavitation conditions MBs rapidly change volume causing their phospholipid monolayer to break up and subsequently release any compounds contained within them into the surrounding tissues. This technology has been developed preclinical for use in targeted delivery in oncology [25] or in disruption of thrombi [26] and there are clinical trials underway.

Not only can MBs potentially be used to track blood supply, but also to deliver a controlled local, non invasive therapeutic intervention at a defined timepoint following a fracture to encourage bone healing, or indeed to treat an already established non‐union.

In this prospective study we have demonstrated that microbubbles reach acute fracture sites in high enough quantities as to allow detection using contrast imaging. This phenomenon has previously been demonstrated by Fisher et al. [27] as a means of determining whether a chronic non‐union was caused by an infection or an atrophic situation however, application in acute fractures remained unexplored. Ultrasound imaging alone has been used as a means of assessing the progression to a union of a fracture site however without the use of contrast agents nor therapeutic intent [12]. Other methods of assessing perfusion within bone have been described, including Magnetic Resonance Imaging (MRI) specifically using gadolinium based contrast. This method has a high degree of 3 dimensional accuracy and spatial resolution, however it is expensive and time consuming, and has little potential to provide a therapeutic delivery technique [28]. Similarly Positron Emission Tomography has been used to assess blood flow, again with variable results. The standard glucose labelled tracers available in day to day practice are unsuitable due to their poor affinity for bone, however fluoride based tracers are available which bind to bone more reliably. However, these are a better marker for bone turnover rather than blood flow. This technique's main limitation however is its expense and radiation exposure in addition to the time consuming nature of the study with peak intensity occurring 45 min or so following injection of contrast agents. Doppler ultrasound alone as a measure of blood flow in fracture healing has been described as a measure of the rate at which fractures are healing, however this technique is technically challenging, difficult to quantitatively assess, and has little in the way of therapeutic potential [29].

A secondary outcome noted in this study is the effect of time from injury on time intensity curve (TIC) analysis parameters. We can see a reduction in the PI with time from fracture. This is matched with a similar decline in AuC. However, this effect is not as clear when looking at the TtP and the difference between WiAUC and WoAUC. There are several possibilities as to why this may be the case. If this effect is simply because the rate of perfusion of the fracture falls off within the first 4 weeks following injury this suggests that previous literature suggesting that peak blood flow occurs 2 weeks after injury is incorrect [11]. Although if this is the case it does call into the question established understanding of perfusion during bone healing. In vivo work has suggested that the peak of canine bone healing blood flow analysed using CEUS may be at around 35 days measured with PI, TtP and AUC following injury, although this was a single animal case report rather than a prospective study [30]. Another possible explanation for this process is that the fracture becomes increasingly echo dense as the density of the callus increases, therefore the signal received from the interaction of microbubbles with the ultrasound beam is attenuated, leaving a reduced signal intensity despite an increasing or at least steady blood flow. This is supported by the fact that the TtP does not appear to reduce relative to time from fracture. This suggests that the rate at which the bubbles are reaching a fracture does not change, merely that the volume of signal detected falls which is in keeping with the argument that the callus density increases and therefore the PI decreases rather than the rate of blood flow.

There are several limitations with this piece of work. This was primarily designed as a pilot study rather than as a significant clinical trial. As such the numbers included are low. Furthermore, there is little known about the validity of amplitude measures across different patients in an acute fracture setting. Other studies have noted high inter observer reliability with other organs such as renal artery [31]. We have tried to minimise any variability by using the same radiologist in all our studies. However it is very difficult to control for factors such as the exact rates of inflow of contrast agent and the exact orientation of the fracture relative to the ultrasound probe. Although none of these should affect the primary aim of determining the presence of microbubbles within the fracture sites. In future studies it may be beneficial to perform serial examinations in the same patient to minimise some of this error. Another potential source of error is the selection of the RoI for the assessment of the contrast intensity. In this study this was done by hand by assessing the B mode imaging to determine the area to be analysed. Other studies have used a similar technique, however not only does this leave a degree of inter and intra observer variability, the video will invariably move a little during the scan and therefore adding a degree of uncertainty. However the fact that the RoI covers the entire visible fracture gap rather than a single point is unlikely that movement or slight inaccuracies should alter the overall results given that the results are averaged across the data capture and that smoothing has been applied to the curve to minimise these effects.

A further questions this study raises is whether the rate of amplitude measures such as PI continues to fall off with time following the fracture. It raises an interesting point as to whether the rate of decline of these measures is associated with whether these fractures go on to unite without any further intervention. Furthermore, the rate of change of these measures in relation to patient comorbidities such as type 2 diabetes will also be of interest.

## 5 Conclusion

This clinical pilot study demonstrates the perfusion of fracture sites using commercially available MBs in acute fracture healing. Though this phenomenon has been observed in established fracture non‐unions this is the first description in the acute healing phase. This demonstrates the potential for using this technology for both diagnostic and therapeutic aims. Further work should focus on longer follow up with serial measurements in a larger cohort of patients to better characterise the changes in observed measurements over the process of fracture healing.

## Author Contributions

D. Cadoux‐Hudson, obtained funding, contributed to study design, collected and analysed data and wrote and revised the manuscript; M. Thomas performed CEUS scans and revised the manuscript; J. Hurst, R. Schranz and A. Gerrish recruited patients and revised the manuscript; K. Wallace analysed the data and revised the manuscript; D. Warwick obtained funding, contributed to study design and revised the manuscript; D. Carugo and E. Stride obtained funding, analysed the data and revised the manuscript; S. Tilley obtained funding, designed the study, analysed data and revised the manuscript; N. D. Evans obtained funding, designed the study, analysed the data and wrote and revised the manuscript.

## Supporting information

Supporting video 1 Patient 2 converted.

Supporting video 2 Patient 1 converted.

Supporting video 3 Patient 3 converted.

Supporting video 4 Patient 4 converted.

Supporting video 5 Patient 5 converted.

Supporting video 6 Patient 6 converted.

Supporting video 7 Patient 7 converted.

Deleted scan (patient 8).

Deleted scan (patient 8) compressed.

JOR70100‐Supporting material files.
