# Supplementary figures and images for: Contrast‐Enhanced Ultrasound (CEUS) Reveals Perfusion of Human Bone in Acute Fracture Healing: A Pilot Study
Source: J Orthop Res. 2025 Dec 13;44(1):e70100. doi: 10.1002/jor.70100 (PMC12701634; doi:10.1002/jor.70100)

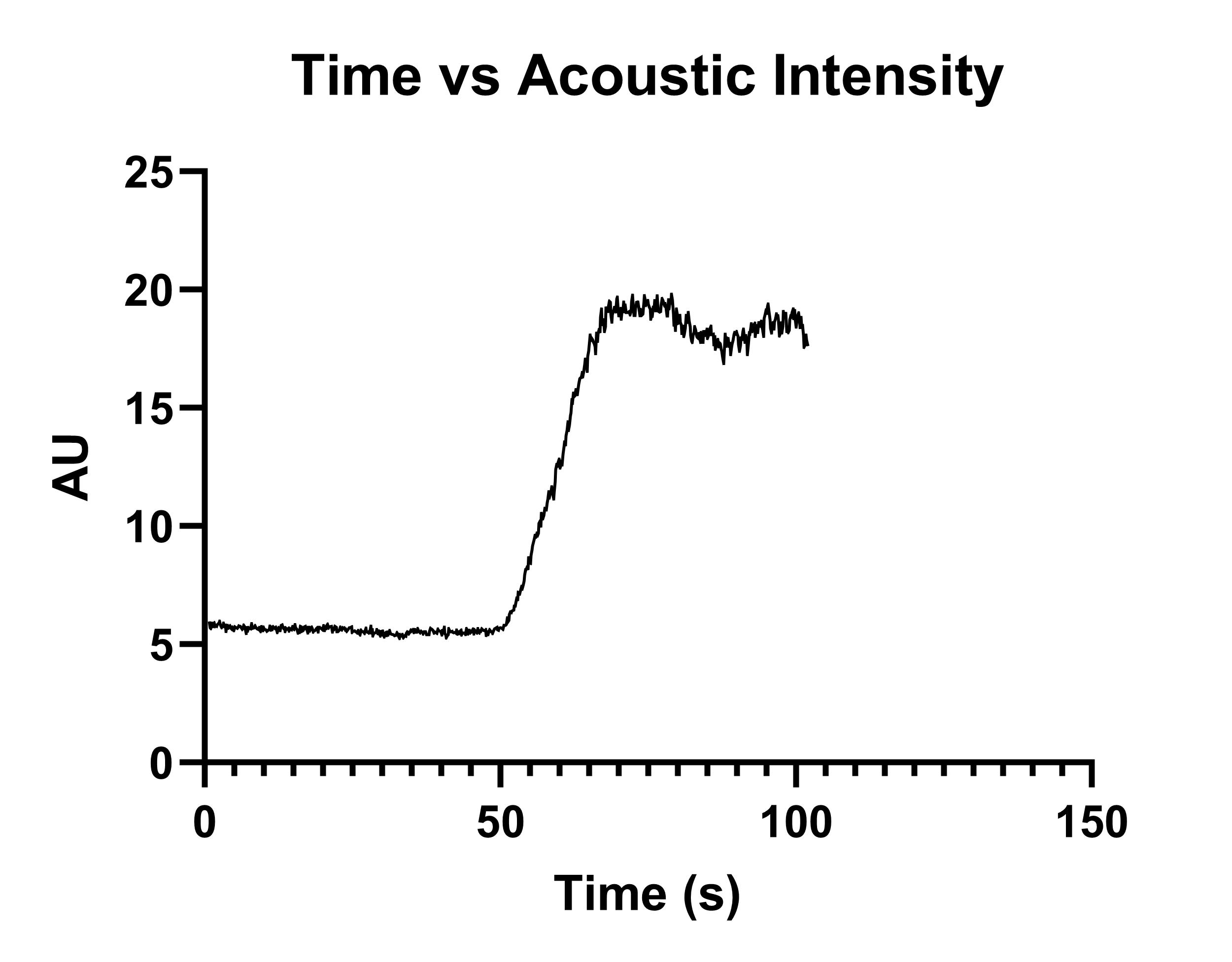

Supplement: Supplementary file 8 — Deleted scan (patient 8). [file JOR-44-0-s007.jpg]
