# Supplementary material for: Contrast‐Enhanced Ultrasound (CEUS) Reveals Perfusion of Human Bone in Acute Fracture Healing: A Pilot Study
Source: J Orthop Res. 2025 Dec 13;44(1):e70100. doi: 10.1002/jor.70100 (PMC12701634; doi:10.1002/jor.70100)
Supplement: Supplementary file 10 — JOR70100‐Supporting material files. [file JOR-44-0-s004.zip › JOR70100-Supplementary material.zip!/SafeSend-aE8mZFJhRqJeP79H/Cadoux-hudson et al Supplementary figures 020924.pdf]

Supplementary Figure 1

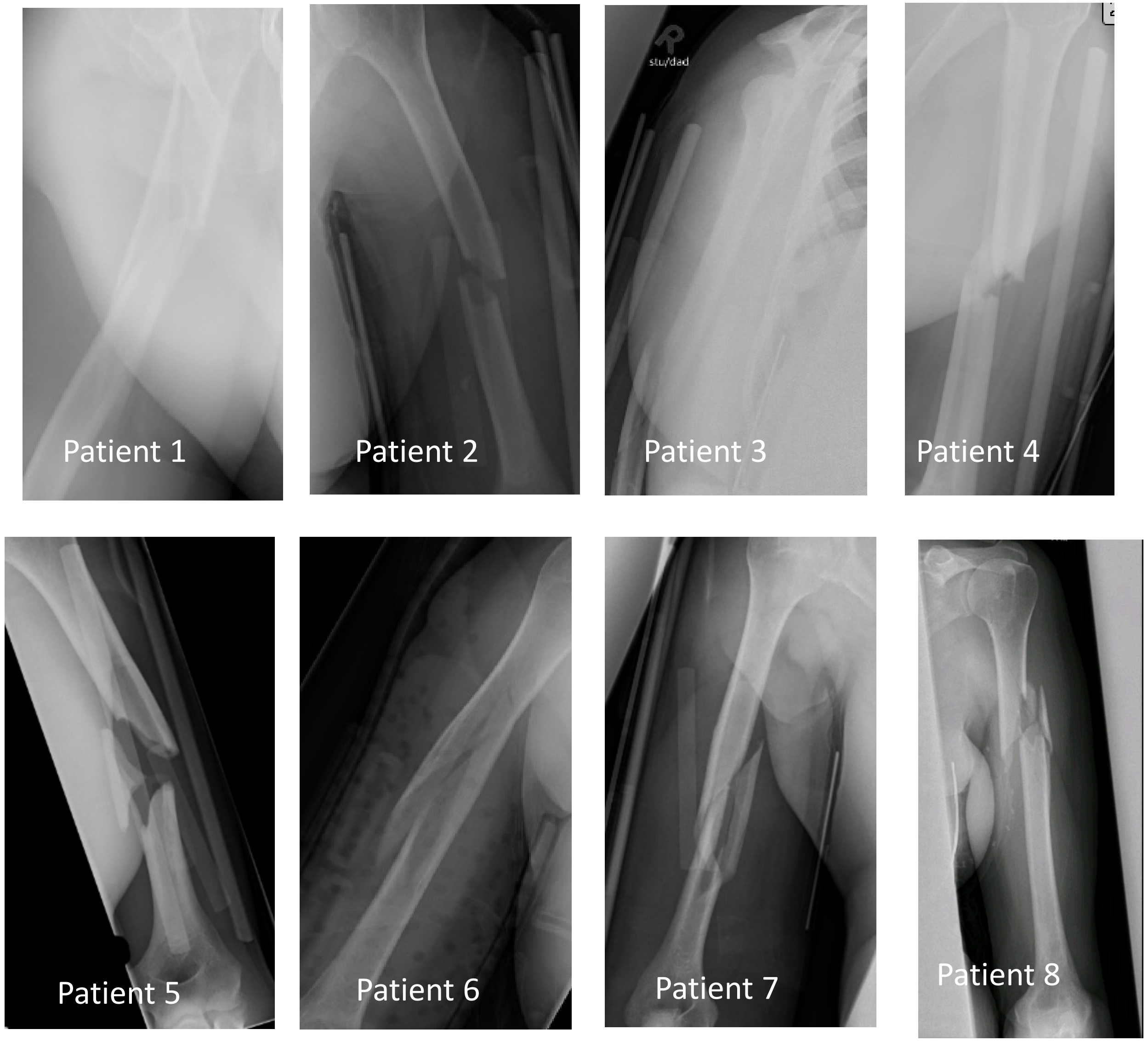

<u>Supp. Fig 1.</u> X ray radiographs for the 8 patients recruited in the trial

Supplementary Figure 2

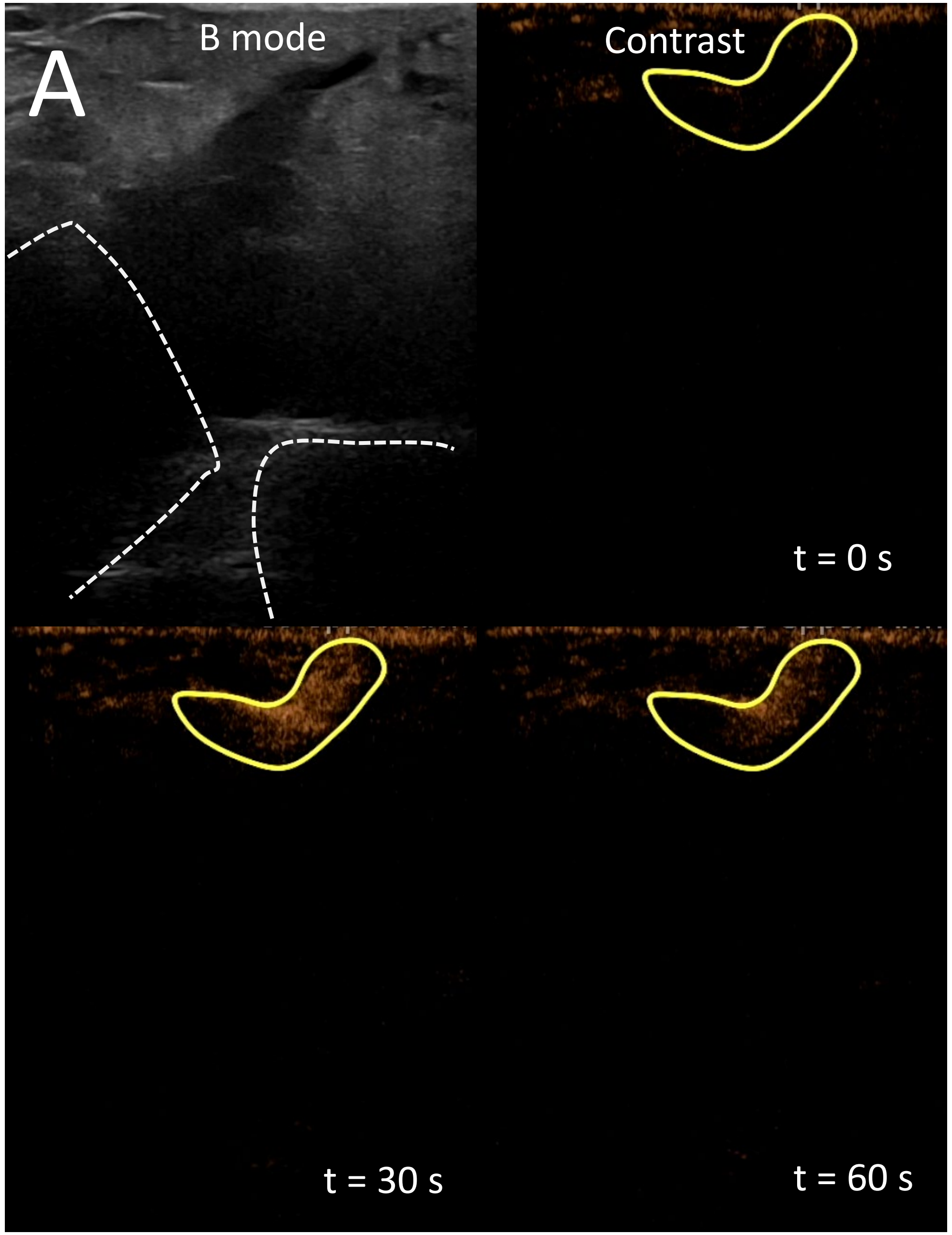

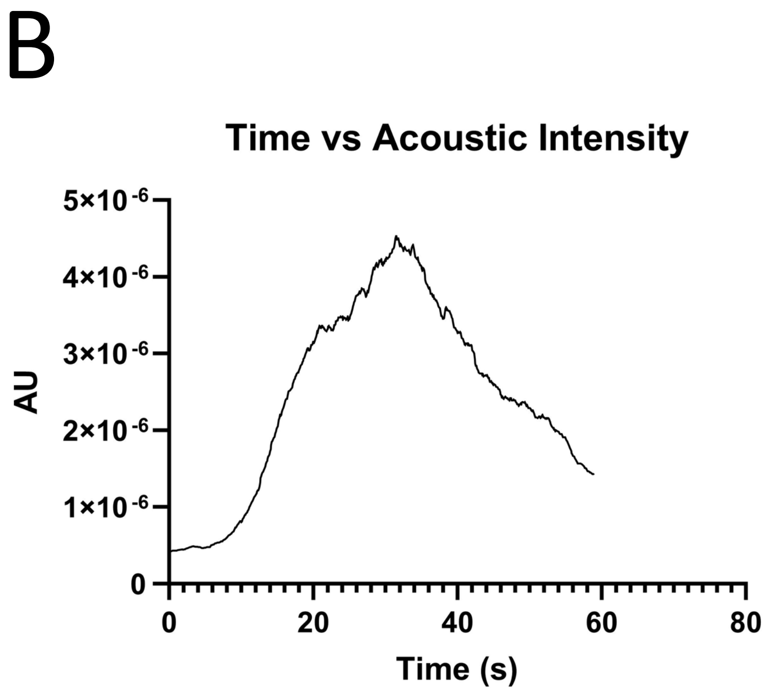

<u>**Supp. Fig 2.**</u> **Dynamic perfusion of fracture gaps can be quantified with respect to time (PATIENT 1).** (A) Fracture regions were marked with a ROI and the relative acoustic intensity was quantified with respect to time (B) showing a rapid peak and a slower wash-out.

Supplementary Figure 3

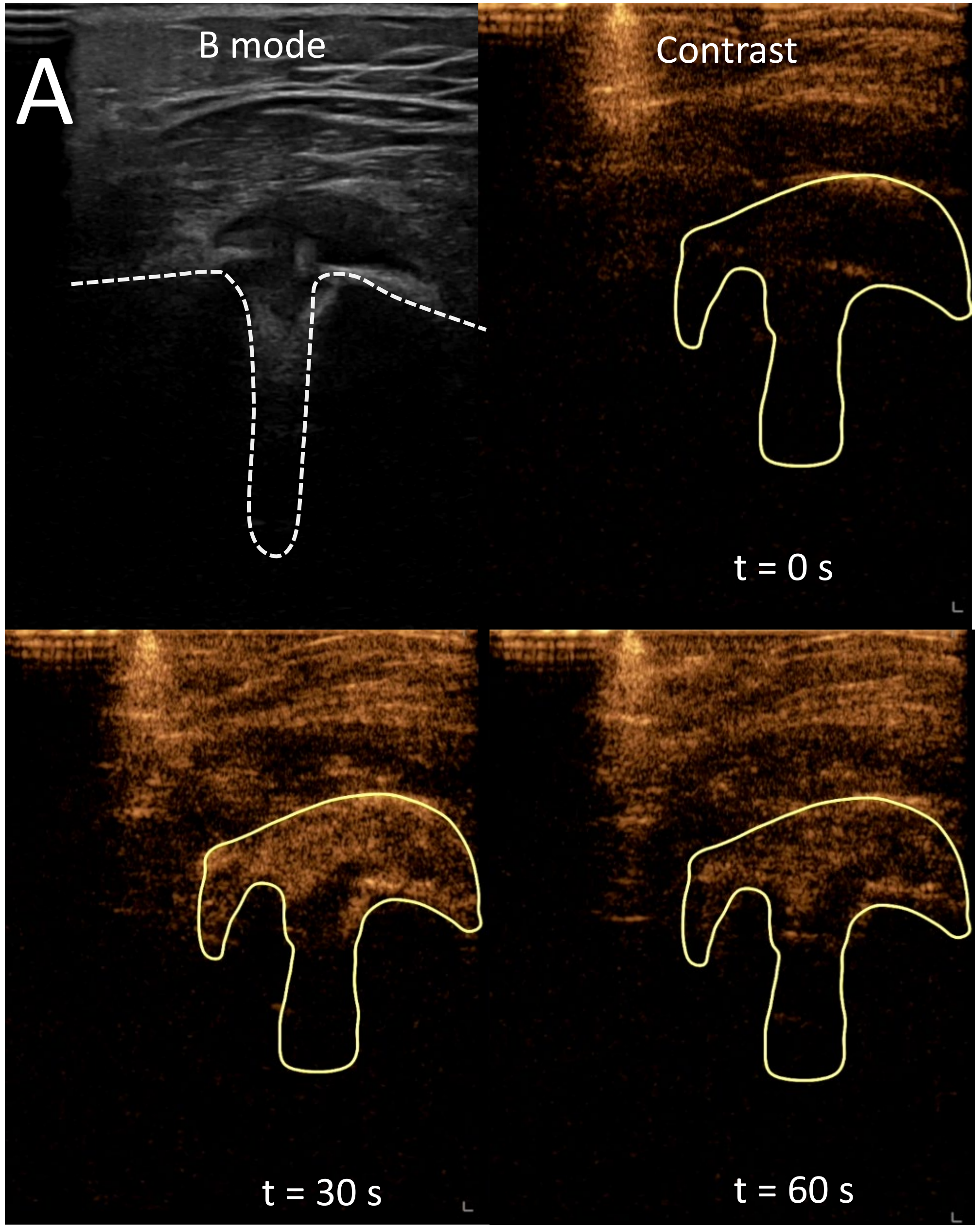

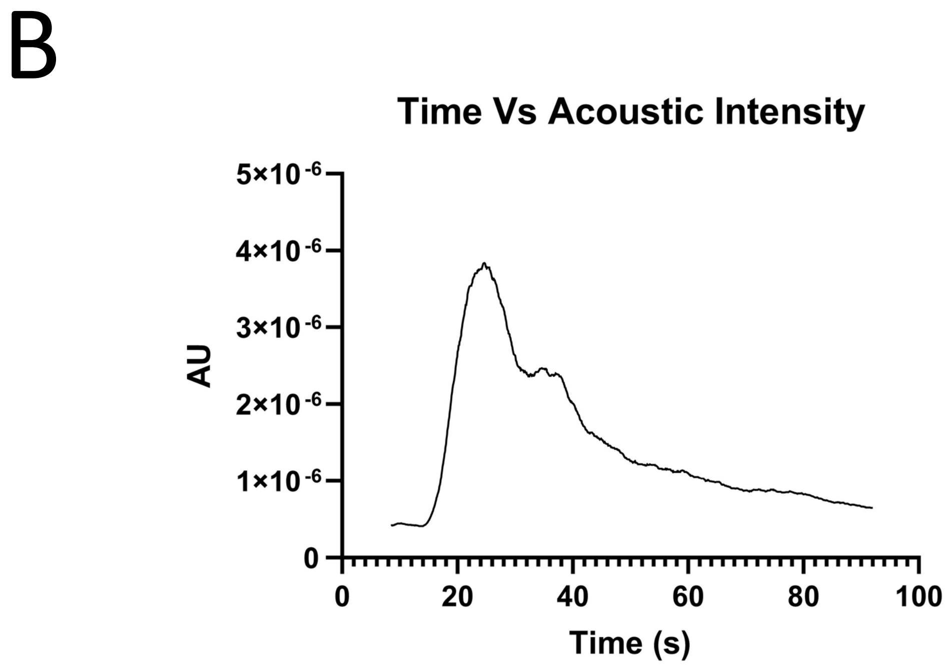

<u>Supp. Fig 3.</u> Dynamic perfusion of fracture gaps can be quantified with respect to time (PATIENT 3). (A) Fracture regions were marked with a ROI and the relative acoustic intensity was quantified with respect to time (B) showing a rapid peak and a slower wash-out.

Supplementary Figure 4

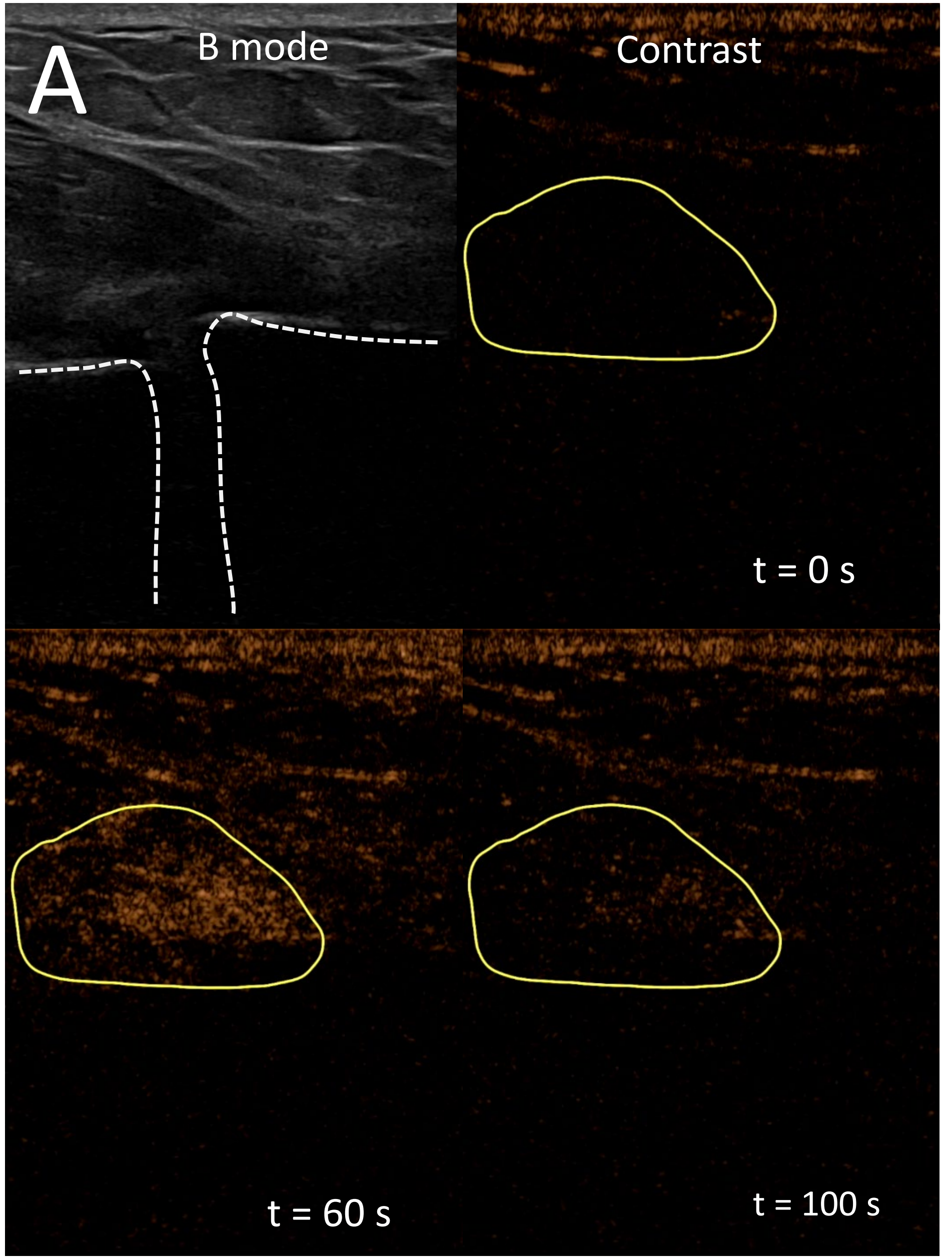

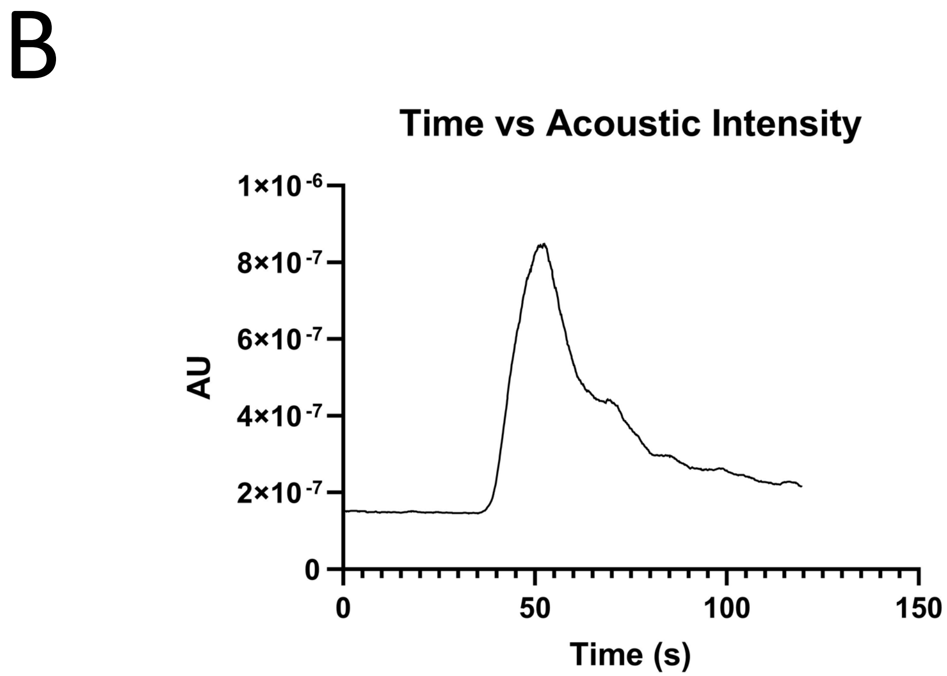

<u>Supp. Fig 4.</u> Dynamic perfusion of fracture gaps can be quantified with respect to time (PATIENT 4). (A) Fracture regions were marked with a ROI and the relative acoustic intensity was quantified with respect to time (B) showing a rapid peak and a slower wash-out.

Supplementary Figure 5

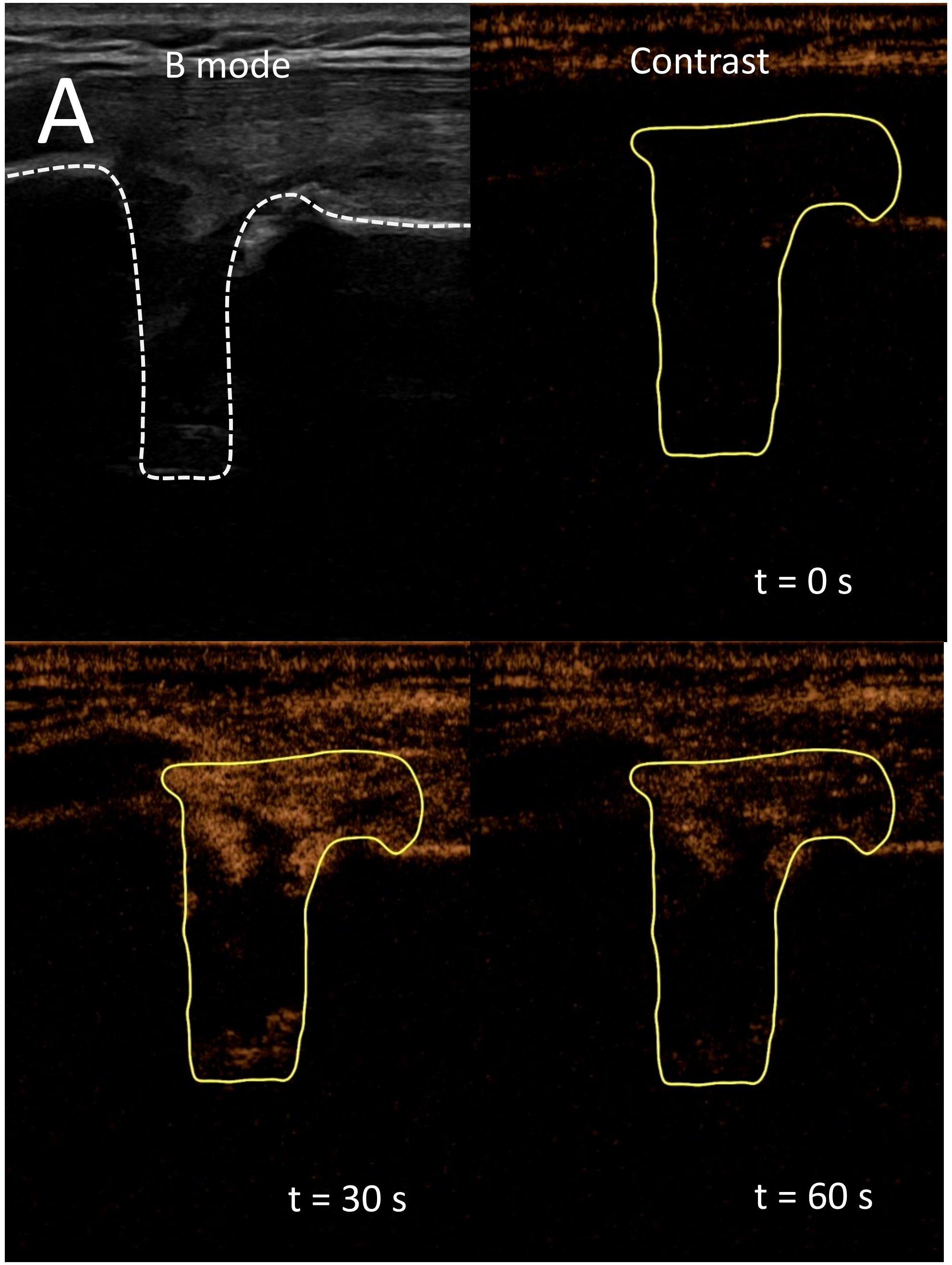

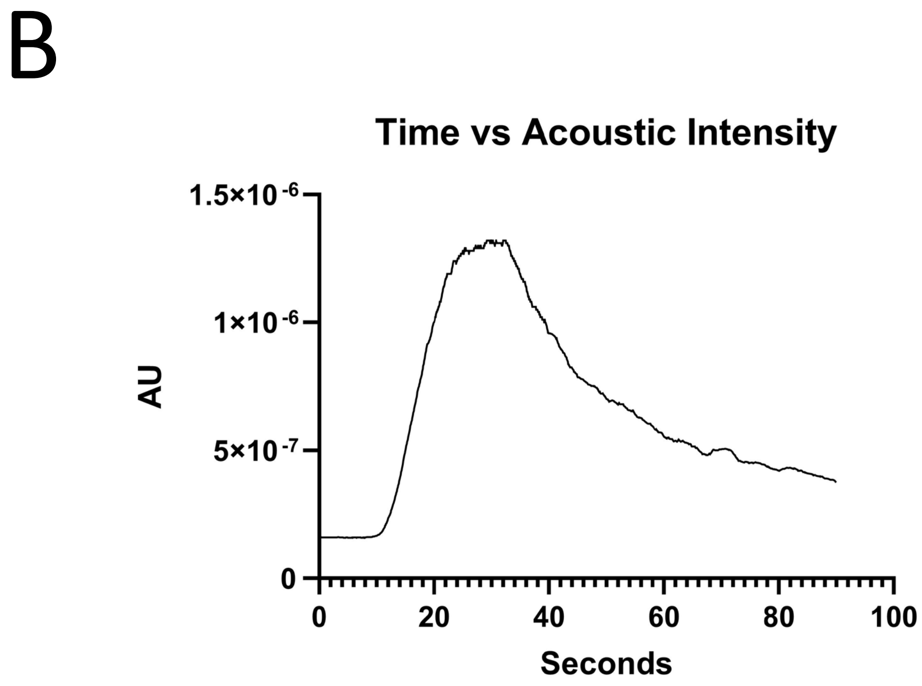

<u>Supp. Fig 5.</u> Dynamic perfusion of fracture gaps can be quantified with respect to time (PATIENT 5). (A) Fracture regions were marked with a ROI and the relative acoustic intensity was quantified with respect to time (B) showing a rapid peak and a slower wash-out.

Supplementary Figure 6

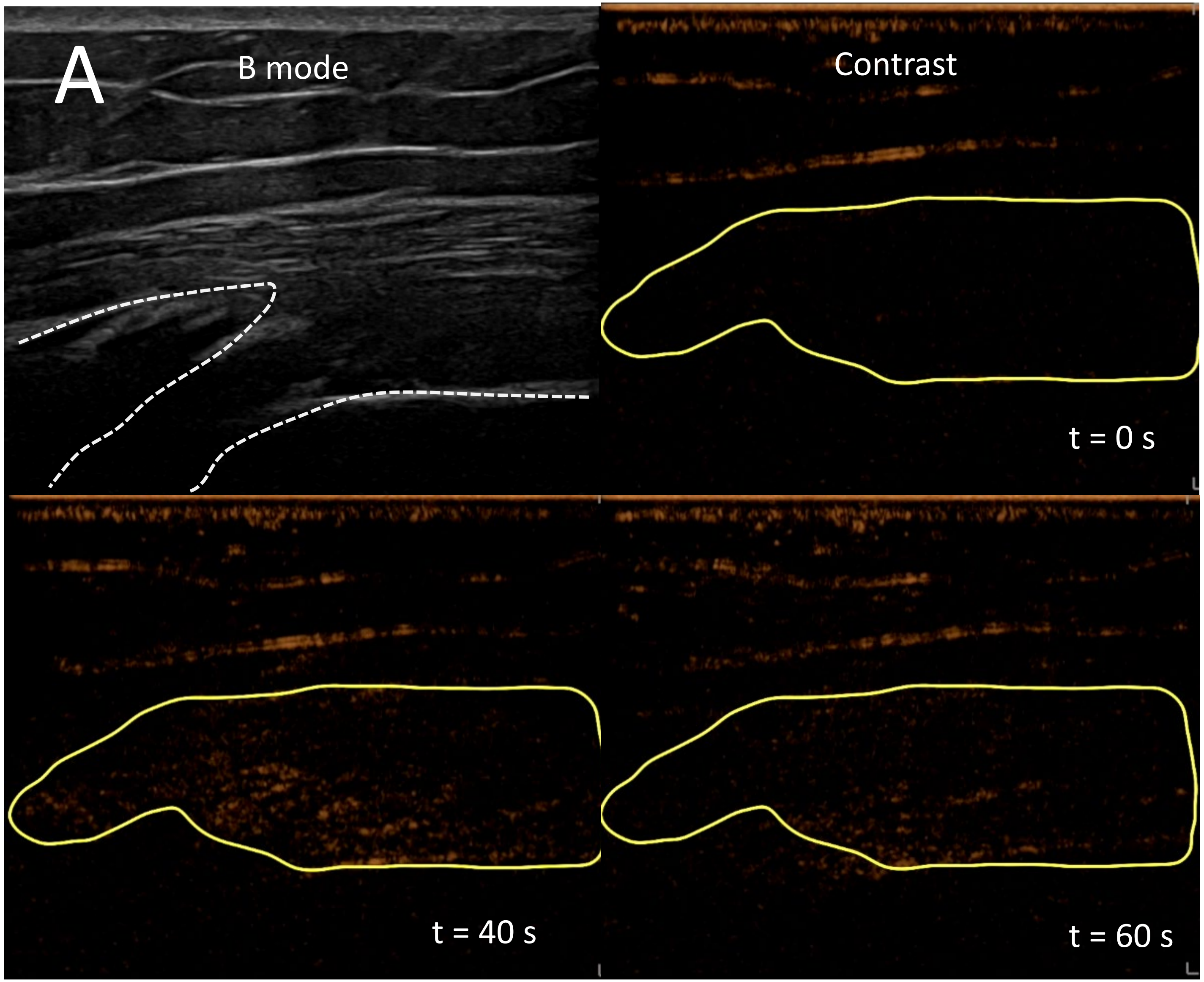

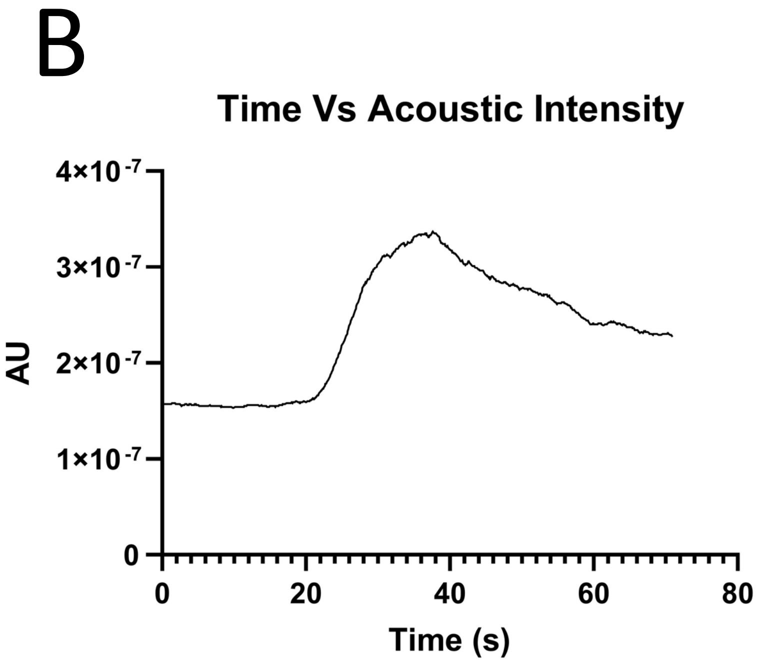

<u>Supp. Fig 6.</u> Dynamic perfusion of fracture gaps can be quantified with respect to time (PATIENT 6). (A) Fracture regions were marked with a ROI and the relative acoustic intensity was quantified with respect to time (B) showing a rapid peak and a slower wash-out.

Supplementary Figure 7

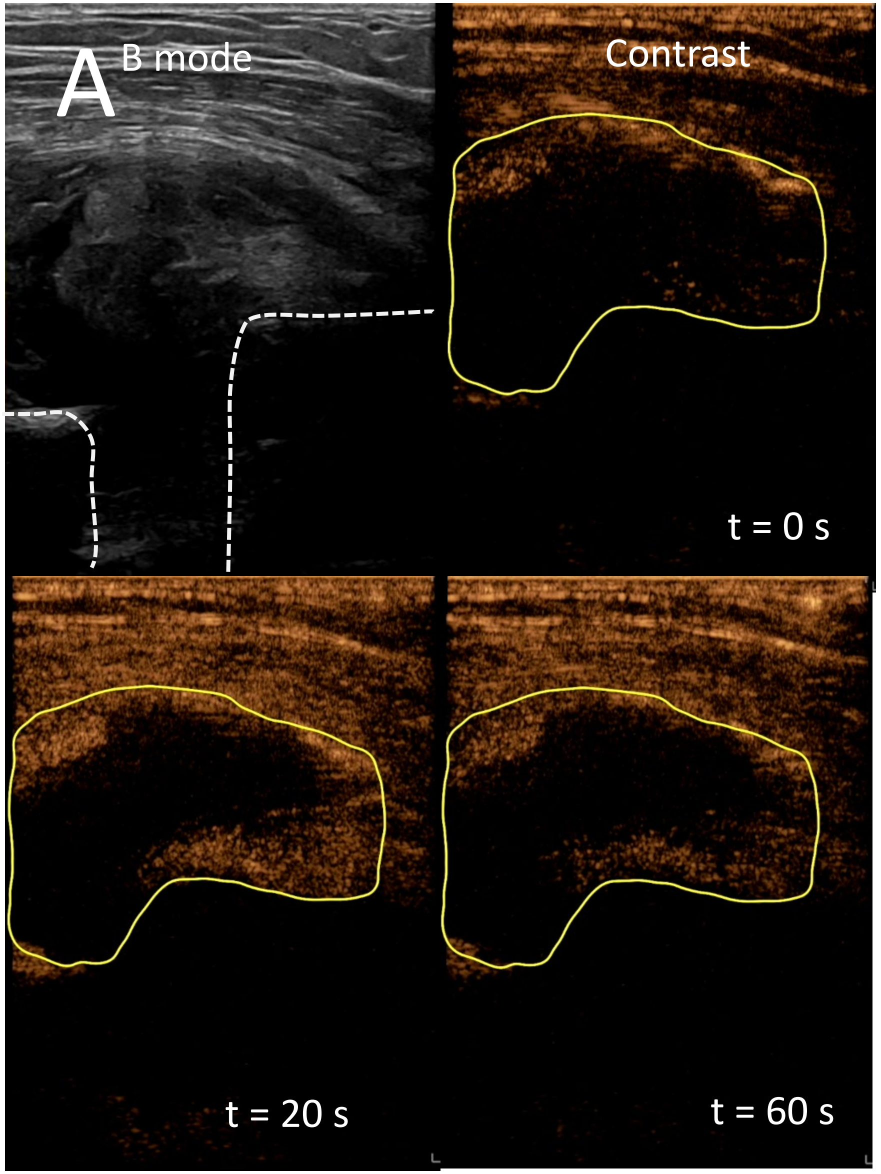

B

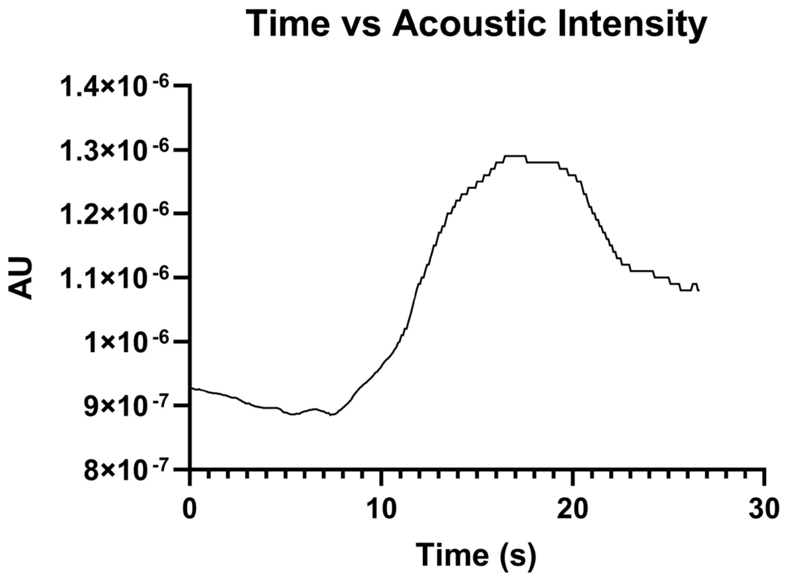

Supp. Fig 7. Dynamic perfusion of fracture gaps can be quantified with respect to time (PATIENT 7). (A) Fracture regions were marked with a ROI and the relative acoustic intensity was quantified with respect to time (B) showing a rapid peak and a slower wash-out.

Supplementary Figure 8

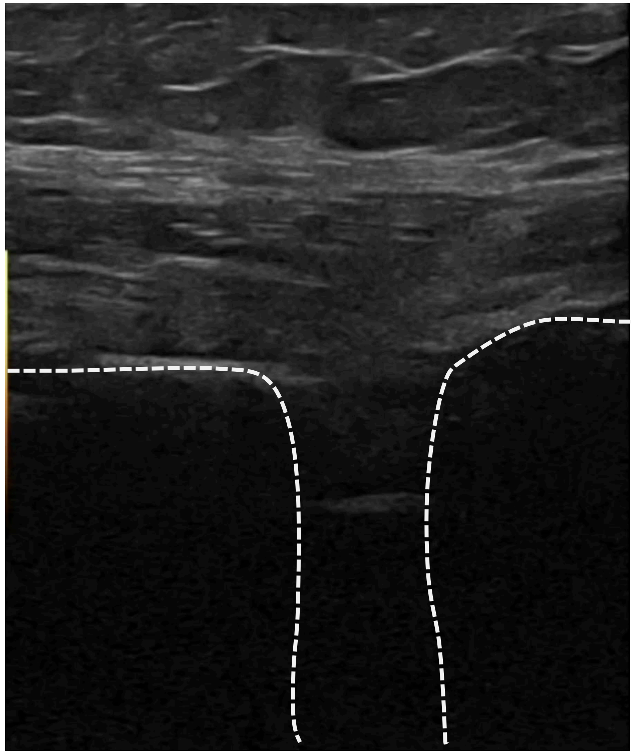

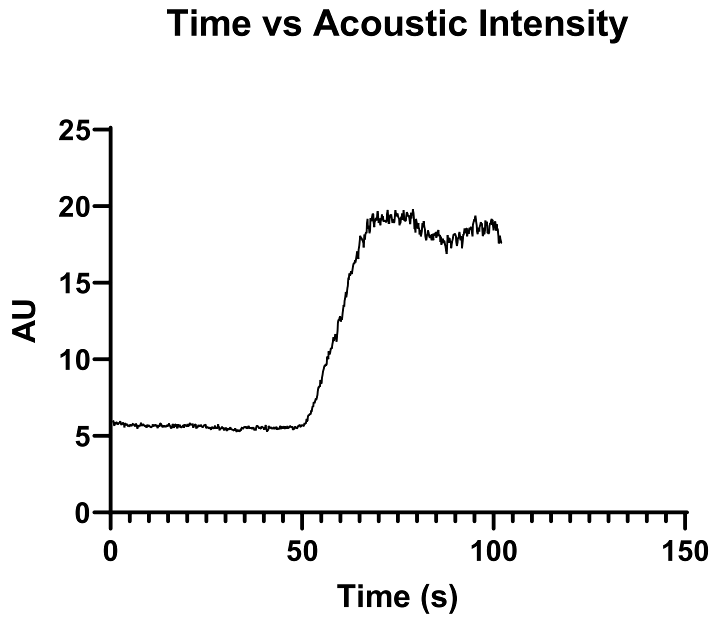

<u>Supp. Fig 8.</u> Dynamic perfusion of fracture gaps can be quantified with respect to time (PATIENT 8). Due to data loss from the Logiq system, the data for patient 8 was obtained using ROI analysis within Image J. A ROI was selected in the same manner as the other patients and analysed using Prism Graphpad software. However the AU used are different and therefore not comparable. There is a similar TIC with a rapid peak and a slow wash out.
