# Supplementary material for: Contrast‐Enhanced Ultrasound (CEUS) Reveals Perfusion of Human Bone in Acute Fracture Healing: A Pilot Study
Source: J Orthop Res. 2025 Dec 13;44(1):e70100. doi: 10.1002/jor.70100 (PMC12701634; doi:10.1002/jor.70100)
Supplement: Supplementary file 10 — JOR70100‐Supporting material files. [file JOR-44-0-s004.zip › JOR70100-Supplementary material.zip!/SafeSend-aE8mZFJhRqJeP79H/Cadoux-Hudson Suppl. Figure Legends.docx]

Cadoux-Hudson et al. *Contrast-enhanced ultrasound (CEUS) reveals perfusion of human bone fracture in acute-phase healing*

**Supplementary Figure Legends**

**Supp. Fig 1.** X ray radiographs for the 8 patients recruited in the trial

**Supp. Fig 2.** **Dynamic perfusion of fracture gaps can be quantified with respect to time (PATIENT 1).** (A) Fracture regions were marked with a ROI and the relative acoustic intensity was quantified with respect to time (B) showing a rapid peak and a slower wash-out.

**Supp. Fig 3.** **Dynamic perfusion of fracture gaps can be quantified with respect to time (PATIENT 3).** (A) Fracture regions were marked with a ROI and the relative acoustic intensity was quantified with respect to time (B) showing a rapid peak and a slower wash-out.

**Supp. Fig 4.** **Dynamic perfusion of fracture gaps can be quantified with respect to time (PATIENT 4).** (A) Fracture regions were marked with a ROI and the relative acoustic intensity was quantified with respect to time (B) showing a rapid peak and a slower wash-out.

**Supp. Fig 5.** **Dynamic perfusion of fracture gaps can be quantified with respect to time (PATIENT 5).** (A) Fracture regions were marked with a ROI and the relative acoustic intensity was quantified with respect to time (B) showing a rapid peak and a slower wash-out.

**Supp. Fig 5.** **Dynamic perfusion of fracture gaps can be quantified with respect to time (PATIENT 5).** (A) Fracture regions were marked with a ROI and the relative acoustic intensity was quantified with respect to time (B) showing a rapid peak and a slower wash-out.

**Supp. Fig 6.** **Dynamic perfusion of fracture gaps can be quantified with respect to time (PATIENT 6).** (A) Fracture regions were marked with a ROI and the relative acoustic intensity was quantified with respect to time (B) showing a rapid peak and a slower wash-out.

**Supp. Fig 7.** **Dynamic perfusion of fracture gaps can be quantified with respect to time (PATIENT 7).** (A) Fracture regions were marked with a ROI and the relative acoustic intensity was quantified with respect to time (B) showing a rapid peak and a slower wash-out.

**Supp. Fig 8.** **Dynamic perfusion of fracture gaps can be quantified with respect to time (PATIENT 8).** Due to data loss from the Logiq system, the data for patient 8 was obtained using ROI analysis within Image J. A ROI was selected in the same manner as the other patients and analysed using Prism Graphpad software. However the AU used are different and therefore not comparable. There is a similar TIC with a rapid peak and a slow wash out.
